# Correction: Influence of micro/nanobubbles on clogging in drip irrigation systems

**DOI:** 10.1039/d0ra90122a

**Published:** 2020-11-20

**Authors:** Hao Li, Hong Li, Qibiao Han, Xiuqiao Huang, Yue Jiang, Hao Sun, Hui Li

**Affiliations:** Research Center of Fluid Machinery Engineering and Technology, Jiangsu University Zhenjiang 212013 China; Institute of Farmland Irrigation, Chinese Academy of Agricultural Sciences Xinxiang 453002 China

## Abstract

Correction for ‘Influence of micro/nanobubbles on clogging in drip irrigation systems’ by Hao Li *et al.*, *RSC Adv.*, 2020, **10**, 38912–38922, DOI: 10.1039/d0ra07782h.

The authors regret the omission of a funding acknowledgement in the original article. This acknowledgement is given below.

**Acknowledgements**

This research was supported by The National Key Research and Development Program of China (2017YFC0403204, 2016YFC0400202) and Central Public-Interest Scientific Institution Basal Research Fund (FIRI202001-01).

The Royal Society of Chemistry apologises for these errors and any consequent inconvenience to authors and readers.

## Supplementary Material

